# A Preliminary Reform Only

**Published:** 1919-02-22

**Authors:** 


### THE NEW NOTIFIABLE DISEASES.

### A Preliminary Reform Only.

On February 14 was published in the *London Gazette* an order making notifiable in England and Wales seven infectious diseases—malaria, dysentery, trench fever, acute pneumonia, enteric fever, relapsing fever, and typhus. As a first step this is more than welcome, but in its present form falls obviously very far short of completeness. In all these conditions, with the exception of pneumonia, the difficulty lies not so much in their control and prevention as in a more essential factor, the diagnosis. One does not doubt that for many home-coming medical men, of temporary foreign and tropical experience, these diseases will hold few mysteries and that other practitioners will rapidly grasp the essential points, but it must be remembered that this new list contains infections hitherto rare in this country and likely now to appear in attenuated, disguised forms often possessed of considerable potential virulence. Ready access to laboratory aid is essential at the outset, for it cannot be suggested that a doctor should perform such technical work himself. Some men at least would be meeting infections they had never before experienced.

There seems to be but one possible method, and that centralisation. Special hospitals or parts of hospitals must be devoted to each disease and thus give added control, efficiency of treatment and, by no means of least importance, opportunity for the instruction of practitioners throughout every district. It is essential that the local doctor should have an exact knowledge of the technique in specimen collection, of precisely what is required and at what clinical phase, while the laboratories themselves must show a maximum development in rapid production of diagnostic reports communicated to both doctor and public authorities. Without all this the scheme may become farcical, for we know that each of these diseases has its own peculiarities in required preventive measures, small perhaps, but in each case vital. Further, in the past, teaching of public health and preventive medicine, except for those who would specialise, has been singularly deficient and accompanied by many unsatisfactory rule-of-thumb methods. Of late ideas have been revolutionised, but though the busy practitioner does his best to keep pace with them it is unfair to lay the diagnostic responsibility on his shoulders, unless some logical means is found to provide first-hand instruction in the new work the Government demands. The country should offer practitioners facilities to gain all the instruction they wish and see that they lose neither time nor money in the attempt to play their part in a great national work. There are frequent rumours that such established provision and payment will ultimately be made through one of the many authorities now in charge of the nation's health, but the time for action is overdue, and further delays must cease. A new and sudden step has been taken, and one can only trust that all the developments it suggests—and indeed requires if it is to prove of any real value—may be rapidly forthcoming.

